# Quantifying serum antibody in bird fanciers' hypersensitivity pneumonitis

**DOI:** 10.1186/1471-2466-6-16

**Published:** 2006-06-26

**Authors:** Charles McSharry, George M Dye, Tengku Ismail, Kenneth Anderson, Elizabeth M Spiers, Gavin Boyd

**Affiliations:** 1 Division of Immunology, Infection and Inflammation, University of Glasgow, Western Infirmary, Glasgow G11 6NT, UK; 2 Department of Immunology, Ninewells Hospital, Dundee DD1 9SY, UK; 3 Department of Respiratory Medicine, Stobhill Hospital, Glasgow G21 3UW, UK; 4 Department of Respiratory Medicine, Crosshouse Hospital, Kilmarnock KA2 0BE, UK

## Abstract

**Background:**

Detecting serum antibody against inhaled antigens is an important diagnostic adjunct for hypersensitivity pneumonitis (HP). We sought to validate a quantitative fluorimetric assay testing serum from bird fanciers.

**Methods:**

Antibody activity was assessed in bird fanciers and control subjects using various avian antigens and serological methods, and the titer was compared with symptoms of HP.

**Results:**

IgG antibody against pigeon serum antigens, quantified by fluorimetry, provided a good discriminator of disease. Levels below 10 mg/L were insignificant, and increasing titers were associated with disease. The assay was unaffected by total IgG, autoantibodies and antibody to dietary hen's egg antigens. Antigens from pigeon serum seem sufficient to recognize immune sensitivity to most common pet avian species. Decreasing antibody titers confirmed antigen avoidance.

**Conclusion:**

Increasing antibody titer reflected the likelihood of HP, and decreasing titers confirmed antigen avoidance. Quantifying antibody was rapid and the increased sensitivity will improve the rate of false-negative reporting and obviate the need for invasive diagnostic procedures. Automated fluorimetry provides a method for the international standardization of HP serology thereby improving quality control and improving its suitability as a diagnostic adjunct.

## Background

Hypersensitivity pneumonitis (HP) is an immune-mediated pulmonary disease caused by repeated inhalation of mainly microbial and animal (glyco-) protein dusts. The most common syndromes are bird fanciers' lung and farmers' lung. HP forms a substantial and important subgroup of interstitial lung diseases [1], which are disorders that primarily affect the lung parenchyma in a diffuse manner [2]. The symptoms include shortness of breath without wheeze, general malaise and aching discomfort in the joints and muscles. There is associated pyrexia with rigors and sweating, and an unproductive cough, which occur generally several hours after exposure to the antigenic dust. Recently it was recommended that the assessment of interstitial lung disease should include testing for antibody against antigens associated with HP [2], however, the diagnosis of HP itself is difficult [3-6]. In a recent review of the diagnostic criteria it was concluded that the presence of serum precipitating antibody was a major diagnostic index [5,6]. Precipitins are a qualitative test and cases of undiagnosed HP have been attributed to improper quality control [3]. In this regard there have been recent recommendations from an HP workshop that diagnostic tests are evaluated [7], and from a national quality assurance scheme that antibody tests be developed that are amenable to standardization [8] and that are quantitative [9]. The aim of this study was to assess various antigens and procedures for quantifying serum antibody in bird fanciers' hypersensitivity pneumonitis.

## Methods

### Study groups: bird fanciers

50 volunteer pigeon fanciers attending a convention were selected for doctor administered structured questionnaire regarding the nature and frequency of symptoms relating to avian exposure. The questionnaire has been validated [10], and subjects were categorized into three groups depending on the likelihood of having HP. Those describing at least 3 respiratory and 3 systemic symptoms occurring together 4–8 hours after pigeon exposure on at least 3 occasions in the last 3 months were categorized as 'Probable' HP [n = 22]. Those with indeterminate symptoms but which did not fulfill the criteria of above were classified as 'Possible' [n = 10], and those with no symptoms associated with pigeon exposure were classified as 'Unlikely' [n = 18] to have HP [10,11]. All were non-smokers.

Subjects signed an informed consent proforma and donated a blood sample. The Medical Ethics Committee of Stobhill Hospital approved the study (ref. 00/44).

Archive sera were available from other bird fanciers who kept the following species: parakeet (budgerigar in UK) (n = 46), cockatiel (n = 13), parrot (n = 11), finch (n = 4), and canary (n = 4).

### Study groups: control subjects

Serum samples were obtained from subjects with no significant contact with pigeons. This included 54 normal healthy adults, and pathological control samples that were selected from archive SLE sera with high levels of auto-antibodies (n = 7), myeloma sera with high levels of total IgG (n = 10), and celiac disease sera with high levels of IgG antibody to hen's egg antigens (n = 32).

### Antigens

Serum from the following avian species; pigeon (*Columbia livia*), finch (*Carduelis sp*.), canary (*Serinus sp*.), cockatiel (*Nymphicus sp*.), parakeet/budgerigar (*Melopsittacus undulatus*), and parrot (*Psittacidae*) was a kind gift of Tom Pennicote, Agricultural Research Station, Auchincruive, Scotland.

### Detection of serum antibody activity against avian antigens

#### Automated fluorimetry

A solid-phase indirect enzyme immuno-assay automated system was used according to the manufacturer's instructions (UniCAP 100, Sweden Diagnostics (UK) Ltd, Milton Keynes, UK). The antigens (code reference) were pigeon serum (Ge93), droppings (e7), and feathers (Re215), and budgerigar serum (e79), feathers (e78), and droppings (e77). When all reagents were in excess, the fluorescence was proportional to the concentration of serum IgG antibody, and the titer was calculated by interpolation onto a 6-point standard curve generated by the fluorescence from standard samples of known IgG titer (traceable to WHO International Reference 67/86).

#### Enzyme immunoassay (EIA)

Serum IgG antibody activity against avian serum antigens was measured by indirect EIA [9]. Optimal conditions established by checkerboard analysis were serum antigen at 5 ug/ml (approximately 1:1000 dilution), and test serum samples diluted at 1:200. The IgG antibody activity was quantified by interpolation, using an optical density standard curve from serial dilutions of a standard serum that had been titrated by quantitative precipitation using pigeon serum gamma globulin antigen [12]. Optical density units were used as a relative measure of the IgG antibody activity against other avian antigens. The specificity of the assay was established by inhibiting the antibody activity in serum with excess free antigen before assay.

#### Precipitin tests for antibody

Serum precipitating antibody against pigeon serum (both Neat and N/10 dilution) was demonstrated by Ouchterlony double diffusion in agar, and the number of precipitin bands that were visible using a microscope eyepiece and indirect lighting was counted.

### Statistical analysis

Results are expressed as median and inter-quartile range unless otherwise specified. Differences between the groups were analyzed using the Mann-Whitney *U*-test and analysis of variance. The IgG antibody titers measured by fluorimetry and by EIA, against pigeon and budgie antigens generated skewed data therefore the correlation coefficient between them was calculated after square root transformation. Statistical significance was accepted at the 95% level.

## Results

### Serum IgG antibody activity against pigeon antigens among pigeon fanciers and control subjects measured by fluorimetry

The titer of serum IgG antibody against antigens derived from pigeon serum, feathers and droppings were significantly higher among 50 pigeon fanciers than 38 control subjects (Table 1, p < 0.001 for all).

The control subjects consisted of 21 healthy subjects and 17 pathological control subjects with either IgG myeloma [n = 10]; to test for the effects of high titers of total IgG on the assay, or SLE [n = 7]; to test for the effects of high titers of polyclonal multi-organ specific auto-antibodies on the assay. The antibody activity in these pathological sera was marginally higher than in the healthy sera for serum and feather antigens (p < 0.01).

### The effect of antibody sensitization to dietary avian antigens on the detection of antibody to inhaled avian antigens

Serum from patients with celiac disease [n = 32] had high titers of IgG to food antigens, including wheat, milk and hen's egg antigens. The serum IgG antibody titer against pigeon serum in these patients was not different from 32 normal healthy serum [mean (SD) ug/ml, 0.24 (0.08), and 0.23 (0.07), respectively, p = 0.88].

### Serum IgG antibody activity against various avian antigens in pigeon fanciers according to symptom category

Among the 50 pigeon fanciers there were 22 with a typical symptom profile of HP [categorized as 'Probable' HP], 10 with indeterminate symptoms ['Possible' HP], and 18 with no significant symptoms caused by avian exposure [categorized as 'Unlikely' to have HP]. The age and gender, and the various avian exposures indices of years keeping pigeons, numbers of pigeons kept and daily hours spent in contact with pigeons were no different between the groups. The serum IgG antibody activity against pigeon serum, droppings and feathers antigens, according to symptom category is listed on Table 2. Those with either 'Probable' or 'Possible' HP could be discriminated from those in the 'Unlikely' category by having significantly higher serum IgG antibody titers against all antigens (p < 0.001 for each). Those with 'Probable' HP could be discriminated from those with 'Possible' HP by having significantly higher antibody titer to antigens from pigeon serum (p = 0.04).

### Antibody activity against different avian species antigens

**Figure 1:**
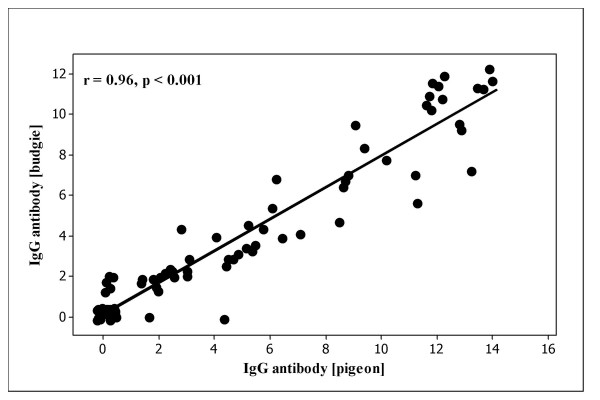
Comparison of the concentrations of serum IgG antibody [ug/ml] against pigeon serum antigens and budgerigar serum quantified by automated fluorimetry in 50 pigeon fanciers. A transformed (square root) scale has been used on both axes.

HP can occur with exposure to many species of pet birds therefore we explored the necessity for using a range of avian species antigens to test for specific immune sensitization. In the first experiment, we compared the antibody activity in 50 pigeon fanciers against either pigeon serum antigens or budgerigar serum antigens (Figure 1). This showed that there was almost equivalent antibody activity detected using either antigen. We then tested the sera of 6 batches of different bird fanciers against serum antigens from each of these different bird species; pigeon, budgerigar, finch, cockatiel, parrot and canary. There was a significant correlation between the antibody activity against each antigen and any of the other antigens (p < 0.001 for all) irrespective of the test antigen or the bird specie to which the subject was exposed. This demonstration of extensive antigenic identity between the avian sera was further confirmed by the demonstration that adding 5 ul of pigeon serum to any subject's serum resulted in >90% inhibition of subsequent detection of antibody activity.

### Antibody activity measured by automated fluorimetry and by EIA

**Figure 2:**
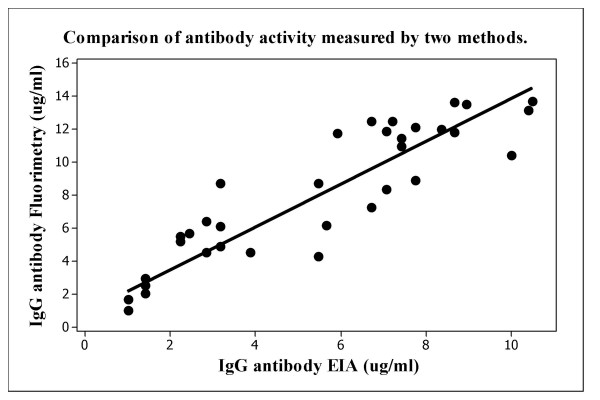
Comparison of the concentrations of serum IgG antibody [ug/ml] against pigeon serum antigens quantified by both in-house enzyme immunoassay [EIA] and by automated fluorimetry in 50 pigeon fanciers. A transformed (square root) scale has been used on both axes.

The IgG antibody against pigeon serum antigens measured by automated fluorimetry and by EIA was compared (Figure 2). Analysis of variance showed that there was no evidence of curvature or a non-zero intercept. Regression analysis demonstrated a correlation coefficient r = 0.923 (p < 0.0001), and the regression equation was: [IgG antibody] **Fluorimeter **= ***a ***[IgG antibody] **EIA**, where the constant of proportionality ***a ***has a value of 0.42 (95% C.I. = 0.31 – 0.54). This suggested that the antibody concentration measured by either technique correlated, but that the antibody concentration measured by EIA was consistently lower than the antibody concentration measured by fluorimetry. The reason for that is because the EIA standard sera were quantified against the gamma-globulin fraction of pigeon serum and would therefore underestimate the antibody activity against whole serum, which will include other major antigens such as albumin.

### Comparison of antibody activity using EIA and precipitin techniques

Precipitin in gel techniques are commonly used to identify antibody as a diagnostic adjunct in HP. In order to identify the detection limit of precipitins we quantified the serum antibody activity against pigeon serum in archived serum from 50 pigeon fanciers by EIA, and compared this with precipitin formation. The antibody titer (mean [SD] ug/ml) of the 31 precipitin-positive subjects was 63 [21] and of the 19 precipitin-negative subjects was 22 [14], (p < 0.001).

### Decrease of the antibody titers after antigen avoidance

**Figure 3:**
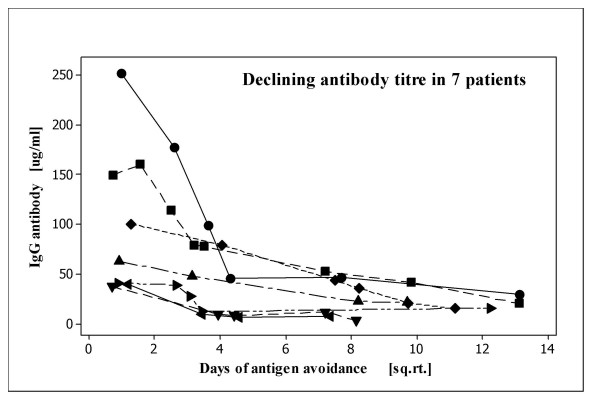
Longitudinal decreasing serum IgG antibody [ug/ml] quantified by EIA in days since the last avian contact in 7 pigeon fanciers shown as individual lines. A transformed (square root) scale has been used on the x-axis.

The longitudinal serum IgG antibody levels against pigeon serum antigens was quantified by EIA in 7 pigeon fanciers who gave up their birds after being diagnosed with HP. Compared with the titer on the first day after removal of antigen, the antibody titers decreased over time is shown on Figure 3. The best estimate of the experimental half-life of this decrease can be calculated from the composite regression equation: Natural log [IgG antibody] = 3.86 - 0.007 days.

This was consistent with an antibody half-life of approximately 95 days when antigen exposure was discontinued. Thus monitoring sequential changes of antibody titer at follow-up clinics was a useful indicator to confirm antigen avoidance.

## Discussion

For the serological investigation of hypersensitivity pneumonitis among bird fanciers we describe the application of an automated standardized fluorometric assay that is unaffected by antibody to dietary avian antigens, multi-organ specific autoantibody and hyper-gammaglobulinaemia. Two recent reports have emphasized the importance of testing for antibody against antigens associated with HP. The Standards of Care Committee of the British Thoracic Society recommended this in the assessment of interstitial lung disease [2], and the international Hypersensitivity Pneumonitis Study Group has confirmed this as an important diagnostic adjunct [6]. We would like to extend this, and recommend that the method used to detect this antibody should be quantitative and amenable to international standardization [8,9].

We suggest that automated fluorimetry provides a suitable method for quantifying serum antibody using the example of bird fanciers' HP. We would recommend an antibody level greater than 10 mg/L as confirmation of significant antigen exposure. This value was based on the upper quartile levels of 8.9 mg/L from pathological control samples, and 11.1 mg/L in the pigeon fanciers who were unlikely to have HP. IgG antibody levels above this we suggest reflect increasing likelihood of HP, for example, pigeon fanciers with symptoms suggesting possible HP had a median level of 70 mg/L, and those with probable HP had a median level of 135 mg/L.

The technical aspects of the fluorimetric assay are important. It is sensitive; with a resolution of 0.1 mg/L, and is linear over several orders of magnitude up to 250 mg/L, which covers the useful dynamic range of antibody titers for this disease [9]. We suggest that pigeon serum is the cleanest and most comprehensive avian antigen for testing sensitivity to most common pet bird species [13]. The assay is robustly standardized against an international IgG standard. It was more sensitive than the more commonly used precipitin formation, which is limited to detecting antibody greater than approximately 40 ug/ml. This increased sensitivity may identify patients with antibody below this level [3,9], with subacute disease or with disease of insidious onset [14], all of which would have been missed by using precipitin formation. Insidious onset is a clinically important subset of HP and these subjects may be represented in our HP 'Unlikely' category because of the lack of acute symptoms. These subjects also tend to have serum antibody titers difficult to detect by precipitins [20], and therefore a more sensitive quantitative assay would be of value for earlier detection to minimize disease progression. This is very important for patients in whom invasive diagnostic procedures including biopsy can be avoided if proper serologic tests are performed using an accurate and reliable reference assay [3,4].

The value of quantifying serum antibody in HP in relation to symptoms is still contentious because many asymptomatic subjects can be sero-positive [15,16]. In cases of antibody positive subjects without symptoms, we and others [14] have found raised levels of serum C-reactive protein, TNF-α and total IgG suggesting active systemic inflammation, as well as physiological evidence of lung inflammation, including increased alveolar permeability [17] and bronchial wall thickness [18]. These observations suggest that the antibody may reflect sub-clinical inflammation, and that individuals only become symptomatic when this inflammation is advanced. This concept is important because HP frequently presents as a chronic disease, and until this hypothesis is resolved it is important to monitor longitudinal changes in titer.

More than 25 million people in USA keep budgerigars, canaries or parrots [19], and budgerigars are the main source of HP in Britain [20]. There are up to 80,000 registered pigeon fanciers in Britain, and there are similar proportions in many other countries worldwide [Dr.P.Lynch, President, British National Flying Club, personal communication], and up to 16% of these can have symptoms in keeping with HP [10-12]. It is important therefore to establish an international epidemiological evidence-base for research into this important disease. Using the methods above it may be possible to provide a standardized approach, and automated fluorimetry will greatly facilitate this in diagnostic laboratories.

## Conclusion

Hypersensitivity Pneumonitis is an important example of Interstitial Lung Disease, and is currently being re-assessed in terms of diagnosis and pathogenesis, for which detecting serum antibody is an important adjunct. There is currently no standardized method for doing this so we propose that an automated and verifiable method be adopted that can be used to compare international studies on diagnosis and pathogenesis. In this pilot study, we describe an automated fluorimetric method that provides for international standardization thereby improving quality control and improving its suitability as a diagnostic adjunct. Using this we find that serum from pigeon is a sufficient antigen to detect antibody in subjects who are exposed to most common pet bird species. We find increasing antibody titer reflected the likelihood of HP, and decreasing titers in longitudinal studies confirmed antigen avoidance. A much larger study will be required to establish the true sensitivity and specificity of quantifying antibody in the diagnosis of HP and we recommend that the first step to achieve this would be to use a standardized method such as automated fluorimetry which could be a reference for all workers in this field. Quantifying antibody was rapid and the increased sensitivity will improve the rate of false-negative reporting and obviate the need for invasive diagnostic procedures.

## Competing interests

The authors have no financial or non-financial competing interests associated with the publication of the manuscript.

## Authors' contributions

All authors have made substantive intellectual contributions to a published study and all have given final approval of the version to be published

**CMS: **Lead the conception and design, performed most of the immunoassays, was responsible for the analysis and interpretation of data; and drafted the manuscript.

**GMD: **Contributed to the conception and design, acquired the automated fluorimetry data, analysed and interpreted this data; and revised the manuscript.

**TI: **Contributed to the conception and design, was involved in the clinical assessment of patients and revised the manuscript.

**KA: **Made substantial contributions to the conception and design, was involved in the clinical assessment of patients and revised the manuscript.

**EMS: **Was responsible for the conception and design, acquisition of automated fluorimetry data, revised the manuscript and was responsible for the acquisition of fluorimetry consumables.

**GB: **Was responsible for the clinical assessment of patients, revised the study critically for important intellectual content and was responsible for the acquisition of funding.

## Pre-publication history

The pre-publication history for this paper can be accessed here:



## Figures and Tables

**Table 1:** Serum IgG antibody (median and i-q range, μg/ml) against pigeon antigens quantified by fluorimetry in pigeon fanciers, and in healthy and pathological control subjects with no significant avian exposure.

	**Pigeon fanciers**	**Control subjects**	
		**Healthy**	**Pathologic**

**Number**	50	21	17
**Pigeon serum**	37.7	0.01	2.5
	*(4.9–133.0)*	*(0.01-0.01)*	*(0.01–8.9)*
**Pigeon feathers**	128.0	0.01	3.8
	*(33.2–250.0)*	*(0.01–2.5)*	*(1.00–4.7)*
**Pigeon droppings**	202.5	21.9	28.2
	*(59.8–250.0)*	*(14.1–26.2)*	*(19.1–42.8)*

**Table 2:** Serum IgG antibody (median and i-q range, μg/ml) against pigeon antigens quantified by fluorimetry in pigeon fanciers according to category of likelihood of Hypersensitivity Pneumonitis, based on symptoms.

	**Category of likelihood of HP**		
	**Probable**	**Possible**	**Unlikely**

**Number**	22	10	18
**Pigeon serum**	135.0	70.3	3.4
	*(49.6–161.0)*	*(23.0–94.9)*	*(0.01–11.1)*
**Pigeon feathers**	250.0	174.0	12.4
	*(142.5–250.0)*	*(80.0–250.0)*	*(2.3–86.4)*
**Pigeon droppings**	250.0	250.0	43.2
	*(250.0-250.0)*	*(121.5–250.0)*	*(26.2–101.8)*
